# Supplementary material for: Molecular determinants of nephron vascular specialization in the kidney
Source: Nat Commun. 2019 Dec 13;10:5705. doi: 10.1038/s41467-019-12872-5 (PMC6910926; doi:10.1038/s41467-019-12872-5)
Supplement: Supplementary file 1 — Supplementary Information [file 41467_2019_12872_MOESM1_ESM.docx]

**Supplementary figure legends**

Molecular determinants of nephron vascular specialization in the kidney

David M. Barry et al.


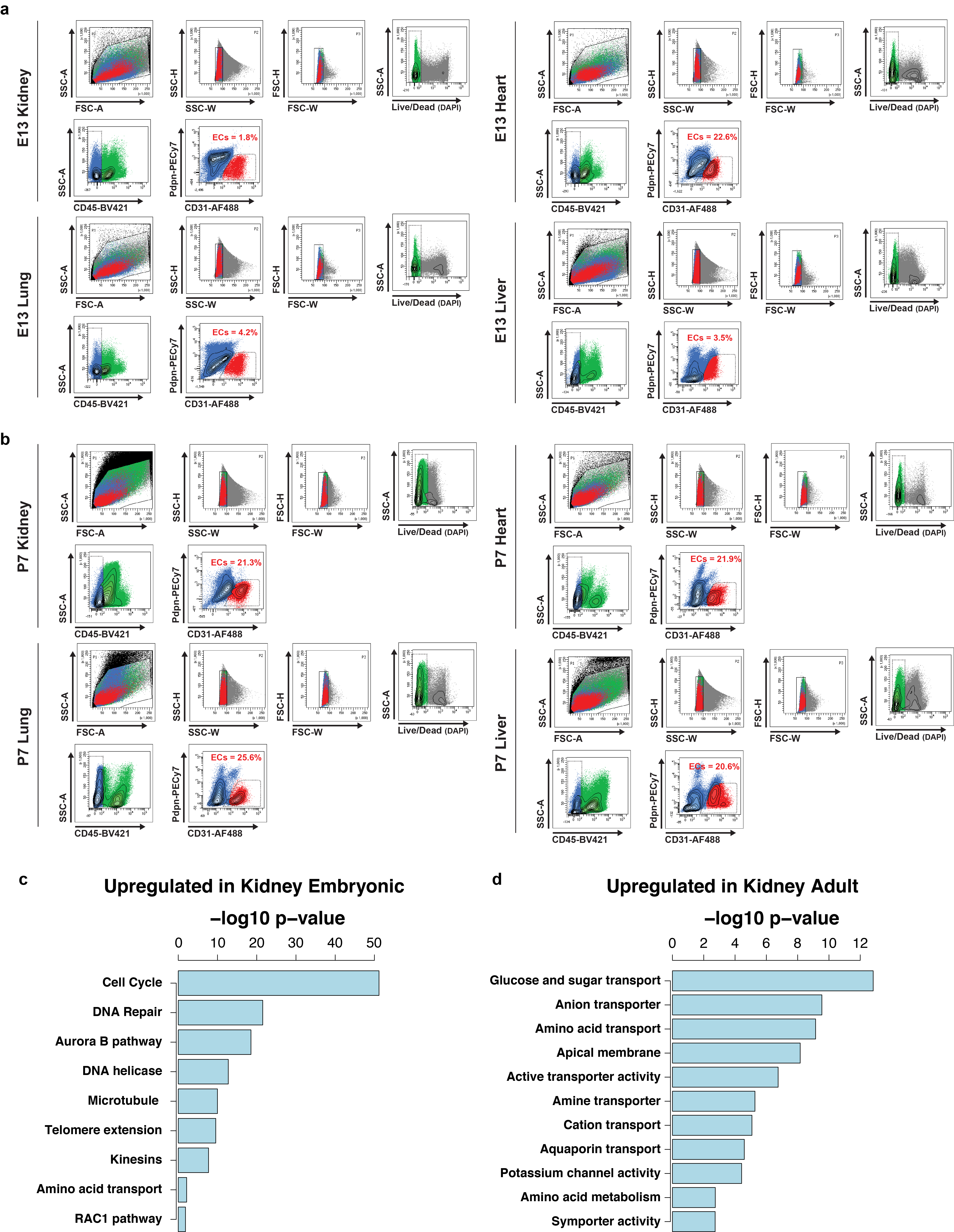


**Supplementary Figure 1: RNA sequencing analysis, related to Figure 1**

a-b) Fluorescent activated cell sorted plots used to isolate endothelial cells (E13 and P7 stages shown).

c-d) –log_10_ p-values for the enrichment of annotated pathways in lists of genes found to be over-expressed (p<.001; FC>2 fold) in the kidney at E13-E17 stages of development (c) or adult stages (d) relative to the heart, liver or lungs.


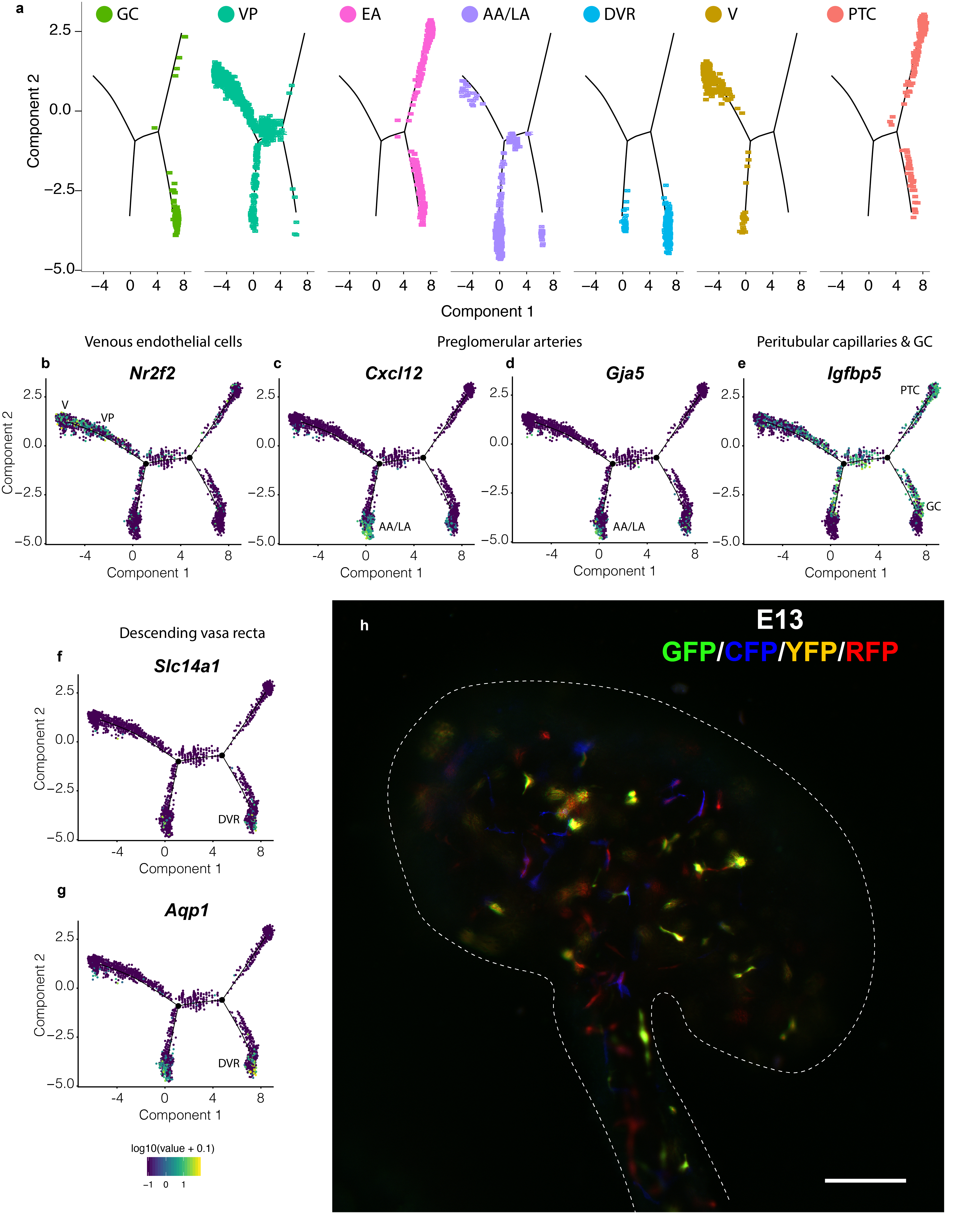


**Supplementary Figure 2: Pseudotime cell fate trajectories, related to Figure 2**

1. Pseudotime trajectory of vascular differentiation in the kidney. Cells corresponding to kidney vascular subtypes are shown here separately. VP, vascular progenitor; PTC, peritubular capillary; AA/LA, afferent arteriole/large arteries (preglomerular); AVR/V, ascending vasa recta/venous blood vessels; EA, efferent arteriole; GC, glomerular capillaries; DVR, descending vasa recta.

b-g) Pseudotime trajectory plots denoting the expression of genes enriched in particular vascular clusters. Plots include Nr2f2 in veins and vascular progenitor cells (b), Cxcl12 and Gja5 in afferent arterioles and other large preglomerular arteries (c-d), Igfbp5 in maturing capillaries and glomeruli (e), and Slc14a1 and Aqp1 in the DVR (f-g).

1. R26R-Confetti E13 kidney pulsed with tamoxifen at E11 (48hrs total, n=4). Single separated cells roughly appeared to be labeled by a single fluorescent reporter. Scale bar 30μm.


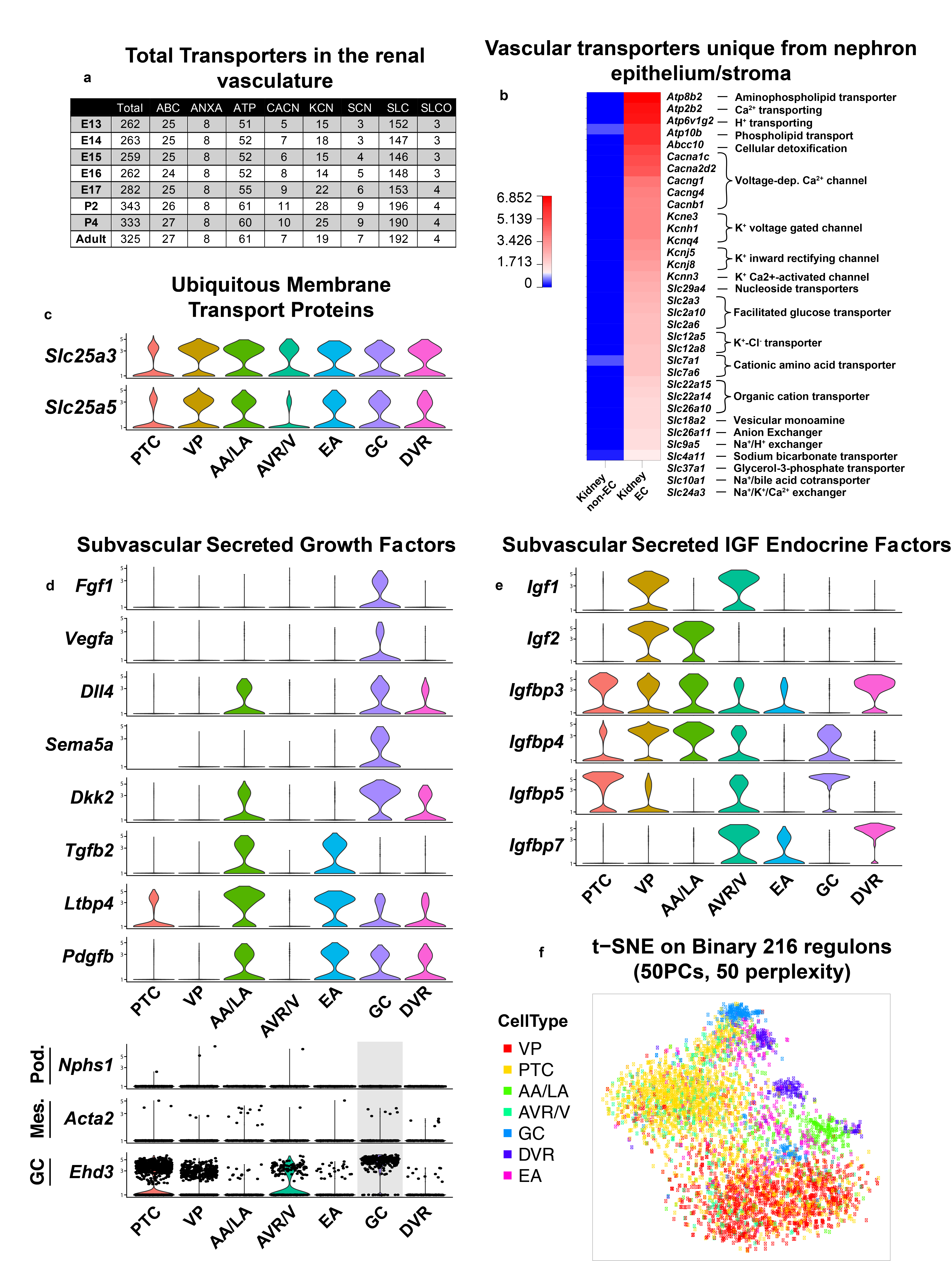


**Supplementary Figure 3: Transcriptional ontology zonation, related to Figure 3**

1. Number of total expressed transporters from each of 7 different transporter classes expressed at each stage of development.
2. Heatmap relating expression of the 34 transporters found to be uniquely expressed in the kidney vasculature in compared to kidney non-endothelial cells.
3. Violin plots of normalized single cell RNA expression profiles denoting ubiquitous membrane transport proteins in the kidney vasculature. Cells are colored according to expression of *Slc25a3* and *Slc25a5.* VP, vascular progenitor; PTC, peritubular capillary; AA/LA, afferent arteriole/large arteries (pre-glomerular); AVR/V, ascending vasa recta/venous blood vessels; EA, efferent arteriole; GC, glomerular capillaries; DVR, descending vasa recta.
4. Violin plots of normalized single-cell RNA expression profiles denoting key kidney subvascular growth factors. Transcripts for *Nphs1* or *Acta2* markers were not present in the glomerular capillary cluster suggesting podocytes or mesangial cells, respectively, are not contaminating the glomerular endothelial cell cluster.
5. Violin plots of normalized single-cell RNA expression profiles denoting IGF family endocrine factors and their regulatory binding proteins.
6. SCENIC results represented after dimensionality reduction tSNE. Endothelial cells were found to similarly generate the kidney vascular subtype clusters described using transcription factor-gene regulons.


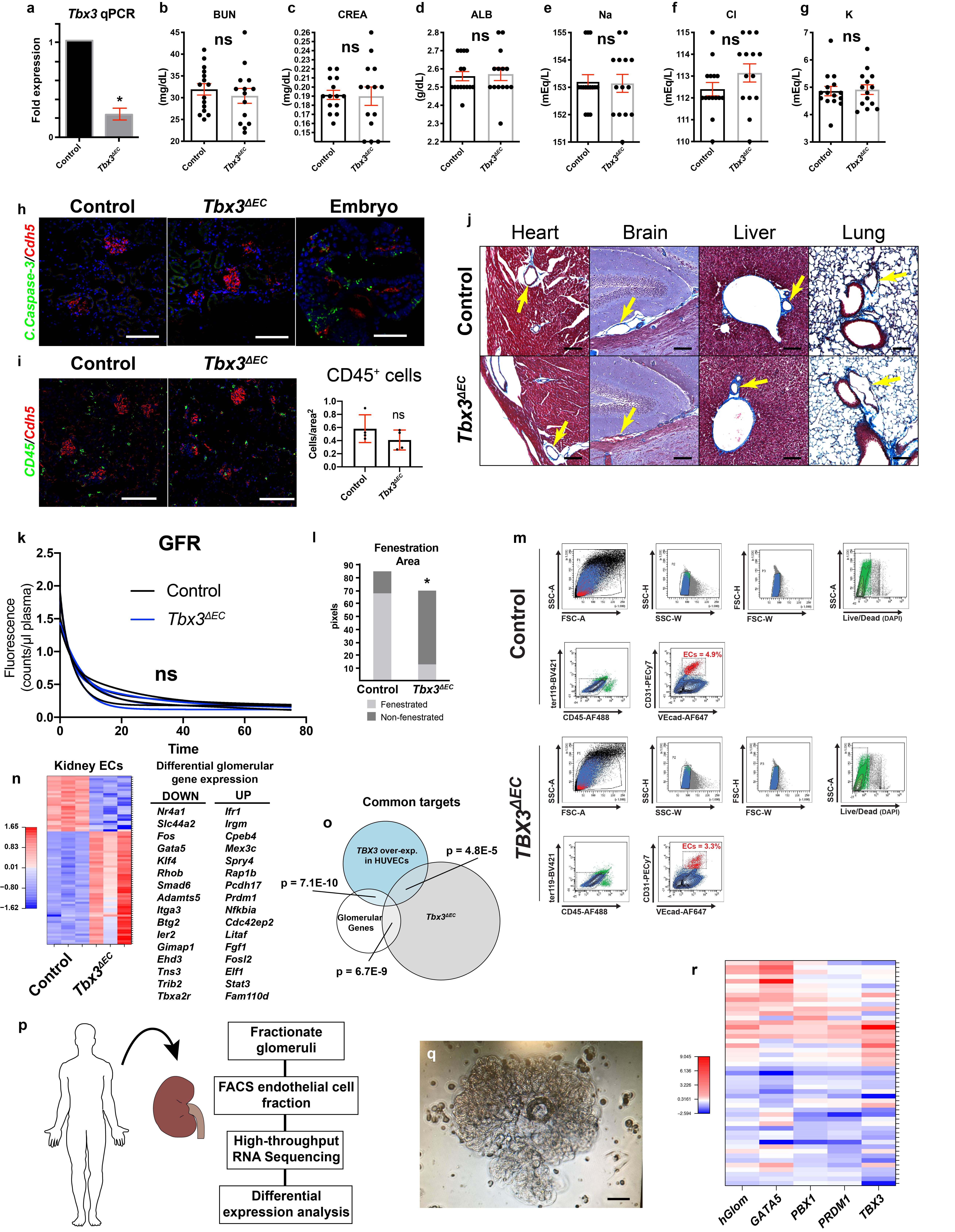


**Supplementary Figure 4: Tbx3 is necessary for GC specification, related to Figure 4**

1. Tbx3 qPCR on Fluorescent activated cell sorted control and *Tbx3^∆EC^* endothelial cells. n = 3. *** =p<.001 unpaired student t-test. Error bars, standard error of the mean.

b-g) Serum analysis panel (control n = 15, *Tbx3^ΔEC^* n = 14). b) Blood urea nitrogen (BUN), c) Creatinine, d) blood albumin (ALB), e) Sodium (Na), f) Chloride (Cl), g) Potassium (K) ns = not significant, * =p<0.05 unpaired student t-test. Error bars, standard error of the mean.

h) Antibody staining of Cleaved Caspase 3 on control and *Tbx3^ΔEC^* mice glomeruli. Natural apoptosis in E9 embryos (positive control). Kidney scale bar 20μm, embryo scale bar 10μm.

1. CD45 antibody staining. Quantification of the stainings. n = 3 mice, 8 fields of view under 40x magnification. ns = not significant, unpaired student t-test. Scale bar 50μm. Error bars, standard error of the mean.
2. Masson’s Trichrome staining of control and *Tbx3^ΔEC^* mice heart, brain, liver, and lung tissue. Yellow arrow indicates arteries. n=4 Control and KO. Scale bar 100μm.
3. Glomerular filtration rate of control and *Tbx3^ΔEC^* mice. n=4 control and KO. ns, not significant, unpaired student t-test.
4. Quantification of the length of fenestrations within glomerular capillaries with microaneurysms. N = 3 kidneys for control and *Tbx3^ΔEC^* mice, 5 fields of view each. * = p < 0.05 unpaired student t-test.
5. Fluorescent activated cell sorted plots used to isolate kidney endothelial cells from control and *Tbx3^ΔEC^* mice.
6. Heatmap relating expression (z-scores) of the genes differentially expressed (p<.05) between control and *Tbx3^∆EC^* endothelial cells (n=3). Select top-ranked genes on right.
7. Euler plot between genes significantly downregulated with Tbx3 overexpression in HUVECs, glomerular specific genes, and genes upregulated in *Tbx3^∆EC^* cells. hypergeometric test p-values for the overlap are indicated.
8. Diagram denoting the workflow to sequence the bulk transcriptome of human glomerular capillaries.
9. Human kidney glomerulus isolated via the glomerular fractionation protocol. Scale bar 20μm.
10. Heatmap representing the top significantly up or downregulated genes in response to each transcription factor (n=3). Human glomeruli (hGlom) are included as a comparator. Values are fold change compared to empty-vector (ALL = collective overexpression of all 4 TF’s)
