# Supplementary material for: Molecular determinants of nephron vascular specialization in the kidney
Source: Nat Commun. 2019 Dec 13;10:5705. doi: 10.1038/s41467-019-12872-5 (PMC6910926; doi:10.1038/s41467-019-12872-5)
Supplement: Supplementary file 3 — Description of Additional Supplementary Files [file 41467_2019_12872_MOESM3_ESM.pdf]

Description of Additional Supplementary Files

File Name: Supplementary Data 1
Description: Normalized counts of the bulk RNAseq data.

File Name: Supplementary Data 2
Description: Differential gene expression among the kidney and heart, liver and lung vasculature for adult and embryonic stages.

File Name: Supplementary Data 3
Description: Raw counts of the single-cell RNA seq data.

File Name: Supplementary Data 4
Description: Normalized counts of the single-cell RNA seq data.

File Name: Supplementary Data 5
Description: Differential expression of genes enriched in different vascular subtypes in the kidney.

File Name: Supplementary Data 6
Description: Profiling of transcription factor, angiocrine factor, and solute transporter gene expression in different kidney vascular subtypes.

File Name: Supplementary Data 7
Description: Differential expression of genes expressed between control and Tbx3ΔEC kidney blood vessels.

File Name: Supplementary Data 8
Description: Differential expression of genes after over expression of control or TBX3, GATA5, PRDM1, PBX1, constructs or all the factors combined in human umbilical vein endothelial cells.

File Name: Supplementary Software 1
Description: SeuratV2 code used for processing and normalizing single cell counts matrices.
