# Supplementary material for: Molecular determinants of nephron vascular specialization in the kidney
Source: Nat Commun. 2019 Dec 13;10:5705. doi: 10.1038/s41467-019-12872-5 (PMC6910926; doi:10.1038/s41467-019-12872-5)
Supplement: Supplementary file 13 — Reporting Summary [file 41467_2019_12872_MOESM13_ESM.pdf]

nature research

Corresponding author(s): David Barry, Shahin Rafii

Reporting Summary

Nature Research wishes to improve the reproducibility of the work that we publish. This form provides structure for consistency and transparency in reporting. For further information on Nature Research policies, see Authors & Referees and the Editorial Policy Checklist.

Statistical parameters

When statistical analyses are reported, confirm that the following items are present in the relevant location (e.g. figure legend, table legend, main text, or Methods section).

n/a	Confirmed	
☐	☒	The exact sample size (n) for each experimental group/condition, given as a discrete number and unit of measurement
☐	☒	An indication of whether measurements were taken from distinct samples or whether the same sample was measured repeatedly
☐	☒	The statistical test(s) used AND whether they are one- or two-sided<br>*Only common tests should be described solely by name; describe more complex techniques in the Methods section.*
☐	☒	A description of all covariates tested
☐	☒	A description of any assumptions or corrections, such as tests of normality and adjustment for multiple comparisons
☐	☒	A full description of the statistics including central tendency (e.g. means) or other basic estimates (e.g. regression coefficient) AND variation (e.g. standard deviation) or associated estimates of uncertainty (e.g. confidence intervals)
☐	☒	For null hypothesis testing, the test statistic (e.g. F, t, r) with confidence intervals, effect sizes, degrees of freedom and P value noted<br>*Give P values as exact values whenever suitable.*
☐	☒	For Bayesian analysis, information on the choice of priors and Markov chain Monte Carlo settings
☐	☒	For hierarchical and complex designs, identification of the appropriate level for tests and full reporting of outcomes
☐	☒	Estimates of effect sizes (e.g. Cohen's d, Pearson's r), indicating how they were calculated
☐	☒	Clearly defined error bars<br>*State explicitly what error bars represent (e.g. SD, SE, CI)*

Our web collection on statistics for biologists may be useful.

Software and code

Policy information about availability of computer code

Data collection	Sequencing data was generated on the Illumina NextSeq500 sequencer.
Data analysis	Raw sequencing data was quality controlled, aligned and count files were generated with FastQC, BBtools, STAR V2.5.3a , samtools V1.5, and HTseq V 0.9.1. Single cell data was processed with the SureCell RNA Single-Cell App in Illumina BaseSpace Sequence Hub. Raw count data was processed with R V.3.4.0

For manuscripts utilizing custom algorithms or software that are central to the research but not yet described in published literature, software must be made available to editors/reviewers upon request. We strongly encourage code deposition in a community repository (e.g. GitHub). See the Nature Research guidelines for submitting code & software for further information.

Data

Policy information about availability of data

All manuscripts must include a data availability statement. This statement should provide the following information, where applicable:

- Accession codes, unique identifiers, or web links for publicly available datasets
- A list of figures that have associated raw data
- A description of any restrictions on data availability

All bulk and single cell expression matrices have been deposited under Gene Expression Omnibus with the accession numbers GSE129005 [https://

April 2018

www.ncbi.nlm.nih.gov/geo/query/acc.cgi?acc=GSE129005] and GSE137786 [https://www.ncbi.nlm.nih.gov/geo/query/acc.cgi?acc=GSE137786]. Other associated raw data are available in Supplementary Data 1-8. All data are available from the corresponding authors upon reasonable request.

Field-specific reporting

Please select the best fit for your research. If you are not sure, read the appropriate sections before making your selection.

- [x] Life sciences
- [ ] Behavioural & social sciences
- [ ] Ecological, evolutionary & environmental sciences

For a reference copy of the document with all sections, see nature.com/authors/policies/ReportingSummary-flat.pdf

Life sciences study design

All studies must disclose on these points even when the disclosure is negative.

Sample size	No sample size calculations were performed for this study. Sample size was determine based on the level and consistency between two different groups.
Data exclusions	Data was excluded for negative data and data that did not have sufficient statistics and is still in progress.
Replication	Results were found consistently at least three times.
Randomization	Mouse litter mates were used and sex matched.
Blinding	Mice were blinded only for blood pressure measurements. All other experiments were sorted by genotype.

Reporting for specific materials, systems and methods

Materials & experimental systems

n/a	Involved in the study	
x		Unique biological materials
	x	Antibodies
	x	Eukaryotic cell lines
x		Palaeontology
	x	Animals and other organisms
	x	Human research participants

Methods

n/a	Involved in the study	
x		ChIP-seq
	x	Flow cytometry
x		MRI-based neuroimaging

Antibodies

Antibodies used

Chicken anti-GFP (for Flk-GFP): Aves, GFP-1020
Goat anti-Cx40/Gja5: Santa Cruz, sc-20466
Rabbit anti-Collagen IV: Millipore, AB756P
Goat anti-Nrp1: R&D Systems, AF566
Rat anti-Plvap: BD Pharmingen, 550563
Goat anti-Sox17: R&D Systems, AF1924
Rat anti-Endomucin: Santa Cruz, sc-65495
Rabbit anti-Aquaporin1: Biorad, MCA2100
Rabbit anti-Tbx3: Abcam, ab99302
Rabbit anti-Aplnr: Protein Tech, 20341-1-AP
Goat anti-Igfbp5: R&D Systems, AF578
Goat anti-Igfbp7: Abcam, ab129302
Rabbit anti-Six2: Protein Tech, 11562-1-AP

Validation

Manufacturer's websites.

Eukaryotic cell lines

Policy information about cell lines

Cell line source(s)	Only primary cells were used. Human umbilical veins endothelial cells and kidney endothelium were isolated from umbilical cords and human kidneys, respectively.
Authentication	The cell lines were not authenticated.
Mycoplasma contamination	Cell lines were not tested for mycoplasma.
Commonly misidentified lines (See ICLAC register)	These lines have not been misidentified in any study as of yet.

Animals and other organisms

Policy information about studies involving animals; ARRIVE guidelines recommended for reporting animal research

Laboratory animals	All animal experiments were performed under the approval of Weill Cornell Medicine Institutional Animal Care and Use Committee (IACUC), New York, NY. The breeding and maintenance of animal colonies abided by the guidelines of the IACUC of Weill Cornell Medical College, New York, New York, USA. All experimental procedures followed the IACUC guidelines. Genotyping was carried out in the laboratory or the tails were sent to Transnetyx (transnetyx.com). To compare the phenotypes between different mouse genotypes, sex- and weight- matched littermates were used. The study used 4 month old male mice.
Wild animals	*Provide details on animals observed in or captured in the field; report species, sex and age where possible. Describe how animals were caught and transported and what happened to captive animals after the study (if killed, explain why and describe method; if released, say where and when) OR state that the study did not involve wild animals.*
Field-collected samples	*For laboratory work with field-collected samples, describe all relevant parameters such as housing, maintenance, temperature, photoperiod and end-of-experiment protocol OR state that the study did not involve samples collected from the field.*

Human research participants

Policy information about studies involving human research participants

Population characteristics	HUVECs were isolated from umbilical cords at the New York Presbyterian Hospital. Human umbilical cords were obtained as left over discarded tissues. The population are healthy full term pregnant woman who have either gone Caesarian section or normal delivery. The permission and active approval for obtaining discarded or left over umbilical cords were obtained from institutional review board (IRB) at Weill Cornell Medicine. The IRB deemed the studies on HUVECs exempt from the requirement of informed consent. The primary HUVECs cultured on plates coated with gelatin in media consisting of M199 (Sigma, M4530), 10% FBS (Omega Scientific, FB07), 50µg ml−1 endothelial mitogen (Alfa Aesar J65416), and 100µg ml−1 heparin (Sigma, H3393).<br><br>Human kidneys used for glomerular endothelial cell isolation or histology were obtained as medical waste from a deceased-donor human kidney that was not transplanted. The deidentified, discarded human kidneys used for research are not considered as "human subject research" as per the standard NIH definition. Hence no IRB approval of the protocol is required. All relevant ethical regulations have been complied with the Institutional Review Board at Weill Cornell Medical College.
Recruitment	The IRB deemed the studies on HUVECs exempt from the requirement of informed consent. As these are discarded tissues the recruitment does not require informed consent and is obtained through the hospital personnel depending on the availability of the discarded and left over umbilical cords.

Flow Cytometry

Plots

Confirm that:

- [x] The axis labels state the marker and fluorochrome used (e.g. CD4-FITC).
- [x] The axis scales are clearly visible. Include numbers along axes only for bottom left plot of group (a 'group' is an analysis of identical markers).
- [x] All plots are contour plots with outliers or pseudocolor plots.
- [x] A numerical value for number of cells or percentage (with statistics) is provided.

Methodology

Sample preparation	To isolate adult endothelial cells from mouse kidney, liver, heart, and lung, mice were injected intravitally with 25µg of anti-VE-cadherin-AF647 antibody (clone BV13, Biolegend) retro-orbitally in 6 to 8-week-old male C57BL/6J mice under anesthesia 10min before they were sacrificed and the organs harvested. For cell sorting, organs were minced and incubated with Collagenase A (25mg ml−1) and Dispase II (25mg ml−1) at 37°C for 20–30min to create a single-cell suspension. Cells were filtered through a 40-µm filter immediately before counter staining. The single-cell suspension was first blocked with an Fc-quenching antibody before antibody staining with anti-mouse CD31-Alexa Fluor® 488 (102414, Biolegend), anti-mouse CD45-Pacific Blue™ (103126,

	Biolegend), and anti-mouse Podoplanin-PE/Cy7(127412, Biolegend). Embryonic tissues were dissected and processes through the same antibodies. Following staining, cells were processed for FACs sorting.
Instrument	BD SORP FACS ARIA2
Software	BD FACSDIVA
Cell population abundance	5-20% of the kidney.
Gating strategy	The single-cell suspension was gated for CD31 488 positive, CD45-Pacific Blue negative, and Podoplanin-PE/Cy7 negative cells.

☒ Tick this box to confirm that a figure exemplifying the gating strategy is provided in the Supplementary Information.
